# Supplementary material for: European Registry on Helicobacter pylori Management: Effectiveness of First and Second-Line Treatment in Spain
Source: Antibiotics (Basel). 2020 Dec 25;10(1):13. doi: 10.3390/antibiotics10010013 (PMC7823881; doi:10.3390/antibiotics10010013)
Supplement: Supplementary file 1 [file antibiotics-10-00013-s001.pdf]

antibiotics

Online Supplementary Material

File S1. Serious adverse events in first– and second–line therapies.

First line

PPI + C + A + M (Conc):

- Esophageal candidiasis and intense abdominal pain with disability (N = 1).
- Intestinal suboclution (N = 1).
- Intense diarrhea with disability (N = 1).

PPI + Single–capsule:

- Acute pancreatitis requiring hospitalization (N = 1).
- Clostridium difficile infection requiring hospitalization (N = 1).

Second line

PPI + L + A:

- Severe diarrhea (N = 1).

N: number of patients presenting the adverse event, PPI: proton pump inhibitor, C: clarithromycin, A: amoxicillin, M: metronidazole, Conc: concomitant administration of the drugs, Single–capsule: three–in–one single capsule, L: levofloxacin.

File S2.Proton pump inhibitor (PPI) categories: low, standard and high acid inhibition.

1. Low dose PPI: ranging from 4.5 to 27 mg omeprazole equivalents, b.i.d. (i.e. 20 mg omeprazole equivalents, b.i.d.). This includes pantoprazole 10 mg, pantoprazole 20 mg, pantoprazole 40 mg, pantoprazole 80 mg, omeprazole 10 mg, omeprazole 20 mg, lansoprazole 15 mg, lansoprazole 30 mg, esomeprazole 10 mg, and rabeprazole 10 mg, all administered b.i.d.
2. Standard dose PPI: ranging from 32 to 40 mg omeprazole equivalents, b.i.d. (i.e. 40 mg

omeprazole equivalents, b.i.d.). This includes omeprazole 40 mg, esomeprazole 20 mg, and rabeprazole 20 mg, all administered b.i.d.

3. High dose PPI: ranging from 54 to 128 mg omeprazole equivalents, b.i.d. (i.e. 60 mg omeprazole equivalents, b.i.d.). This includes omeprazole 80 mg, lansoprazole 60 mg, esomeprazole 40 mg, esomeprazole 80 mg, and rabeprazole 40 mg, all administered b.i.d.

File S3. Contribution log.

María Caldas. Hospital Universitario de La Princesa, Instituto de Investigación Sanitaria Princesa (IIS–IP), Universidad Autónoma de Madrid (UAM) and Centro de Investigación Biomédica en Red de Enfermedades Hepáticas y Digestivas (CIBERehd), Madrid, SPAIN. Supervised, collected data, performed the data quality review, analysed and interpreted the data, wrote the manuscript drafts and approved the submitted manuscript.

Olga P. Nyssen. Hospital Universitario de La Princesa, Instituto de Investigación Sanitaria Princesa (IIS–IP), Universidad Autónoma de Madrid (UAM) and Centro de Investigación Biomédica en Red de Enfermedades Hepáticas y Digestivas (CIBERehd), Madrid, SPAIN. Scientific Director and member of the project's Scientific Committee, planned and coordinated the study, designed and programmed the electronic case report form, performed the data quality review, analysed and interpreted the data, critically reviewed the manuscript drafts, and approved the submitted manuscript.

Ignasi Puig. Althaia Xarxa Assistencial Universitària de Manresa and Universitat de Vic–Universitat Central de Catalunya (UVicUCC), Manresa, SPAIN. Scientific Director and member of the project's Scientific Committee, critically reviewed the manuscript drafts, and approved the submitted manuscript.

Francis Megraud. Laboratoire de Bactériologie, Hôpital Pellegrin, Bordeaux, FRANCE. French National Coordinator and Member of the project's Scientific Committee, designed the protocol, planned the study, critically reviewed the manuscript drafts, and approved the submitted manuscript.

Colm O´Morain. Department of Clinical Medicine,Trinity College Dublin, Dublin, IRELAND. Irish National Coordinator and Member of the project's Scientific Committee, designed the protocol, planned the study, collected data, critically reviewed the manuscript drafts, and approved the submitted manuscript.

Javier P. Gisbert. Hospital Universitario de La Princesa, Instituto de Investigación Sanitaria Princesa (IIS–IP), Universidad Autónoma de Madrid (UAM) and Centro de Investigación Biomédica en Red de Enfermedades Hepáticas y Digestivas (CIBERehd), Madrid, SPAIN. Directed the project and the project's Scientific Committee, acted as Spanish National Coordinator, obtained funding, designed the protocol and planned the study, collected patients, analysed and interpreted the data, critically reviewed the manuscript drafts, and approved the final submitted manuscript.

Ángeles Pérez–Aisa. Agencia Sanitaria Costa del Sol, Red de Investigación en Servicios de Salud en Enfermedades Crónicas (REDISSEC), Marbella, SPAIN. Collected and helped interpreting data, critically reviewed the manuscript drafts, and approved the submitted manuscript.

Nuria Fernández. Agencia Sanitaria Costa del Sol, Red de Investigación en Servicios de Salud en Enfermedades Crónicas (REDISSEC), Marbella, SPAIN. Acquired data, critically reviewed the manuscript drafts, and approved the submitted manuscript.

Inmaculada Santaella. Agencia Sanitaria Costa del Sol, Red de Investigación en Servicios de Salud en Enfermedades Crónicas (REDISSEC), Marbella, SPAIN. Acquired data, critically reviewed the manuscript drafts, and approved the submitted manuscript.

Manuel Castro–Fernández. Hospital de Valme, Sevilla, SPAIN. Collected and helped interpreting data, critically reviewed the manuscript drafts, and approved the submitted manuscript.

Alma Keco Huerga. Hospital de Valme, Sevilla, SPAIN. Acquired data, critically reviewed the manuscript drafts, and approved the submitted manuscript.

Manuel Pabón–Carrasco. Centro Universitario de Cruz Roja, Universidad de Sevilla, Sevilla, SPAIN. Collected and helped interpreting data, critically reviewed the manuscript drafts, and approved the submitted manuscript.

Ana Garre. Hospital Universitario de La Princesa, Instituto de Investigación Sanitaria Princesa (IIS–IP), Universidad Autónoma de Madrid (UAM) and Centro de Investigación Biomédica en Red de Enfermedades Hepáticas y Digestivas (CIBERehd), Madrid, SPAIN, Collected and helped interpreting data, critically reviewed the manuscript drafts, and approved the submitted manuscript.

Alicia C. Marín. Hospital Universitario de La Princesa, Instituto de Investigación Sanitaria Princesa (IIS–IP), Universidad Autónoma de Madrid (UAM) and Centro de Investigación Biomédica en Red de Enfermedades Hepáticas y Digestivas (CIBERehd), Madrid, SPAIN. Acquired data, critically reviewed the manuscript drafts, and approved the submitted manuscript.

Almudena Durán. Hospital Universitario de La Princesa, Instituto de Investigación Sanitaria Princesa (IIS–IP), Universidad Autónoma de Madrid (UAM) and Centro de Investigación Biomédica en Red de Enfermedades Hepáticas y Digestivas (CIBERehd), Madrid, SPAIN. Acquired data, critically reviewed the manuscript drafts, and approved the submitted manuscript.

Jennifer Fernandez Pacheco. Hospital Universitario de La Princesa, Instituto de Investigación Sanitaria Princesa (IIS–IP), Universidad Autónoma de Madrid (UAM) and Centro de Investigación Biomédica en Red de Enfermedades Hepáticas y Digestivas (CIBERehd), Madrid, SPAIN. Acquired data, critically reviewed the manuscript drafts, and approved the submitted manuscript.

Luis Bujanda. Hospital Donostia/Instituto Biodonostia and CIBERehd, Universidad del País Vasco (UPV/EHU), San Sebastián, SPAIN. Collected and helped interpreting data, critically reviewed the manuscript drafts, and approved the submitted manuscript.

Ángel Cosme. Hospital Donostia/Instituto Biodonostia and CIBERehd, Universidad del País Vasco (UPV/EHU), San Sebastián, SPAIN. Collected and helped interpreting data, critically reviewed the manuscript drafts, and approved the submitted manuscript.

Alfredo J. Lucendo. Hospital General de Tomelloso and CIBERehd, Ciudad Real, SPAIN. Collected and helped interpreting data, critically reviewed the manuscript drafts, and approved the submitted manuscript.

Teresa Angueira. Hospital General de Tomelloso and CIBERehd, Ciudad Real, SPAIN. Collected and helped interpreting data, critically reviewed the manuscript drafts, and approved the submitted manuscript.

Luis Rodrigo. Hospital Central de Asturias, Oviedo, SPAIN. Collected and helped interpreting data, critically reviewed the manuscript drafts, and approved the submitted manuscript.

Jose M. Huguet. Consorcio Hospital General Universitario de Valencia, Valencia, SPAIN. Collected and helped interpreting data, critically reviewed the manuscript drafts, and approved the submitted manuscript.

Enrique Medina. Consorcio Hospital General Universitario de Valencia, Valencia, SPAIN. Acquired data, critically reviewed the manuscript drafts, and approved the submitted manuscript.

Luis Ferrer. Consorcio Hospital General Universitario de Valencia, Valencia, SPAIN. Acquired data, critically reviewed the manuscript drafts, and approved the submitted manuscript.

Pilar Canelles. Consorcio Hospital General Universitario de Valencia, Valencia, SPAIN. Acquired data, critically reviewed the manuscript drafts, and approved the submitted manuscript.

Jorge Pérez–Lasala. HM Sanchinarro, Madrid, SPAIN. Collected and helped interpreting data, critically reviewed the manuscript drafts, and approved the submitted manuscript.

Javier Molina–Infante. Hospital San Pedro de Alcántara and CIBERehd, Cáceres, SPAIN. Collected and helped interpreting data, critically reviewed the manuscript drafts, and approved the submitted manuscript.

Pilar Mata–Romero. Hospital San Pedro de Alcántara and CIBERehd, Cáceres, SPAIN. Collected and helped interpreting data, critically reviewed the manuscript drafts, and approved the submitted manuscript.

Jesús Barrio. Hospital Universitario Río Hortega, Valladolid, SPAIN. Collected and helped interpreting data, critically reviewed the manuscript drafts, and approved the submitted manuscript.

Luis Fernández–Salazar. Hospital Clínico Universitario de Valladolid, Valladolid, SPAIN. Collected and helped interpreting data, critically reviewed the manuscript drafts, and approved the submitted manuscript.

Noelia Alcaide. Hospital Clínico Universitario de Valladolid, Valladolid, SPAIN. Collected and helped interpreting data, critically reviewed the manuscript drafts, and approved the submitted manuscript.

Benito Velayos. Hospital Clínico Universitario de Valladolid, Valladolid, SPAIN. Acquired data, critically reviewed the manuscript drafts, and approved the submitted manuscript.

Ángel Lanas. Hospital Clínico Universitario Lozano Blesa and CIBERehd, Zaragoza, SPAIN. Collected and helped interpreting data, critically reviewed the manuscript drafts, and approved the submitted manuscript.

Inés Ariño. Hospital Clínico Universitario Lozano Blesa and CIBERehd, Zaragoza, SPAIN. Collected and helped interpreting data, critically reviewed the manuscript drafts, and approved the submitted manuscript.

Mónica Perona. Hospital Quirón Marbella, Málaga, SPAIN. Collected and helped interpreting data, critically reviewed the manuscript drafts, and approved the submitted manuscript.

Manuel Domínguez–Cajal. Hospital General San Jorge, Huesca, SPAIN. Collected and helped interpreting data, critically reviewed the manuscript drafts, and approved the submitted manuscript.

Juan Ortuño. Hospital Universitari y Politècnic La Fe de Valencia and CIBERehd, Valencia, SPAIN. Collected and helped interpreting data, critically reviewed the manuscript drafts, and approved the submitted manuscript.

Tommaso Di Maira. Hospital Universitari y Politècnic La Fe de Valencia and CIBERehd, Valencia, SPAIN. Collected and helped interpreting data, critically reviewed the manuscript drafts, and approved the submitted manuscript.

Blas José Gómez–Rodríguez. Hospital Universitario Virgen Macarena, Sevilla, SPAIN. Collected and helped interpreting data, critically reviewed the manuscript drafts, and approved the submitted manuscript.

Manuel Rodriguez–Tellez. Hospital Universitario Virgen Macarena, Sevilla, SPAIN. Acquired the data, critically reviewed the manuscript drafts, and approved the submitted manuscript.

Pedro Almela. Hospital General Universitario de Castellón, Castellón de la Plana, SPAIN. Collected and helped interpreting data, critically reviewed the manuscript drafts, and approved the submitted manuscript.

Marina Roldán Lafuente. Hospital General Universitario de Castellón, Castellón de la Plana, SPAIN. Acquired data, critically reviewed the manuscript drafts, and approved the submitted manuscript.

Josep María Botargués. Hospital Universitari de Bellvitge, Barcelona, SPAIN. Collected and helped interpreting data, critically reviewed the manuscript drafts, and approved the submitted manuscript.

Oscar Núñez. Hospital Universitario Sanitas La Moraleja, Madrid, SPAIN. Collected and helped interpreting data, critically reviewed the manuscript drafts, and approved the submitted manuscript.

Inés Modolell. Consorci Sanitari de Terrassa, Barcelona, SPAIN. Collected and helped interpreting data, critically reviewed the manuscript drafts, and approved the submitted manuscript.

Judith Gómez. Complejo Asistencial Universitario de Burgos, Burgos, SPAIN. Collected and helped interpreting data, critically reviewed the manuscript drafts, and approved the submitted manuscript.

Manuel Alfonso Jimenez Moreno. Complejo Asistencial Universitario de Burgos, Burgos, SPAIN. Acquired data, critically reviewed the manuscripts' drafts, and approved the submitted manuscript.

Rafael Ruiz–Zorrilla. Hospital Sierrallana, Cantabria, SPAIN. Collected and helped interpreting data, critically reviewed the manuscript drafts, and approved the submitted manuscript.

Cristóbal de la Coba. Hospital de Cabueñes, Gijón, SPAIN. Collected and helped interpreting data, critically reviewed the manuscript drafts, and approved the submitted manuscript.

Pilar Varela. Hospital de Cabueñes, Gijón, SPAIN. Acquired data, critically reviewed the manuscript drafts, and approved the submitted manuscript.

Alain Huerta. Hospital de Galdakao–Usansolo, Vizcaya, SPAIN. Collected and helped interpreting data, critically reviewed the manuscript drafts, and approved the submitted manuscript.

Eduardo Iyo. Hospital Comarcal de Inca, Mallorca, SPAIN. Collected and helped interpreting data, critically reviewed the manuscript drafts, and approved the submitted manuscript.

Liliana Pozzati. Hospital de Mérida, Badajoz, SPAIN. Collected and helped interpreting data, critically reviewed the manuscript drafts, and approved the submitted manuscript.

Rosario Antón. Hospital Clínic Universitari de Vàlencia, Valencia, SPAIN. Collected and helped interpreting data, critically reviewed the manuscript drafts, and approved the submitted manuscript.

Mercé Barenys. Hospital de Viladecans, Barcelona, SPAIN. Collected and helped interpreting data, critically reviewed the manuscript drafts, and approved the submitted manuscript.

Miguel Fernández–Bermejo. Clínica San Francisco, Cáceres, SPAIN. Collected and helped interpreting data, critically reviewed the manuscript drafts, and approved the submitted manuscript.

Ana Campillo. Hospital Reina Sofía, Tudela, Navarra, SPAIN. Collected and helped interpreting data, critically reviewed the manuscript drafts, and approved the submitted manuscript.

Javier Alcedo. Hospital de Barbastro, Huesca, SPAIN. Collected and helped interpreting data, critically reviewed the manuscript drafts, and approved the submitted manuscript.

Ramón Pajares–Villaroya. Hospital Infanta Sofía, Madrid, SPAIN. Collected and helped interpreting data, critically reviewed the manuscript drafts, and approved the submitted manuscript.

Marianela Mego. Hospital Universitario General de Catalunya, Barcelona, SPAIN. Collected and helped interpreting data, critically reviewed the manuscript drafts, and approved the submitted manuscript.

Fernando Bermejo. Hospital Universitario de Fuenlabrada. Instituto de Investigación Sanitaria Hospital La Paz (IdiPaz), Madrid, SPAIN. Collected and helped interpreting data, critically reviewed the manuscript drafts, and approved the submitted manuscript.

Alicia Algaba. Hospital Universitario de Fuenlabrada. Instituto de Investigación Sanitaria Hospital La Paz (IdiPaz), Madrid, SPAIN. Acquired data, critically reviewed the manuscript drafts, and approved the submitted manuscript.

Jose Luis Dominguez–Jiménez. Hospital Alto del Guadalquivir, Jaén, SPAIN. Collected and helped interpreting data, critically reviewed the manuscript drafts, and approved the submitted manuscript.

Llucia Titó. Hospital de Mataró, Barcelona, SPAIN. Collected and helped interpreting data, critically reviewed the manuscript drafts, and approved the submitted manuscript.

Bárbara Gómez. Hospital de Mataró, Barcelona, SPAIN. Acquired data, critically reviewed the manuscript drafts, and approved the submitted manuscript.

Judith Sánchez Millastre. Hospital Miguel Servet, Zaragoza, SPAIN. Acquired data, critically reviewed the manuscript drafts, and approved the submitted manuscript.

Pilar Sánchez–Pobre. Hospital Doce de Octubre, Madrid, SPAIN. Acquired data, critically reviewed the manuscript drafts, and approved the submitted manuscript.

Fermin Estremera. Complejo Hospitalario de Navarra, Pamplona, SPAIN. Acquired data, critically reviewed the manuscript drafts, and approved the submitted manuscript.

Edurne Amorena. Complejo Hospitalario de Navarra, Pamplona, SPAIN. Acquired data, critically reviewed the manuscript drafts, and approved the submitted manuscript.

Albert Tomàs. Consorci Sanitari del Garraf, Sant Pere de Ribes, Barcelona, SPAIN. Acquired data, critically reviewed the manuscript drafts, and approved the submitted manuscript.

Francisco José Martínez Cerejo. Hospital Universitari Sant Joan de Reus SPAIN Acquired the data, critically reviewed the manuscript drafts, and approved the submitted manuscript.

Xavier Calvet. Corporació Sanitària Universitària Parc Taulí. CIBERehd, Instituto de Salud Carlos III Departament de Medicina. Universitat Autònoma de Barcelona, Sabadell, SPAIN. Acquired data, critically reviewed the manuscript drafts, and approved the submitted manuscript.

Ariadna Figuerola. Corporació Sanitària Universitària Parc Taulí. CIBERehd, Instituto de Salud Carlos III Departament de Medicina. Universitat Autònoma de Barcelona, Sabadell, SPAIN. Acquired data, critically reviewed the manuscript drafts, and approved the submitted manuscript.

Diego Burgos Santamaría. Hospital Universitario Ramón y Cajal, Madrid, SPAIN. Acquired data, critically reviewed the manuscript drafts, and approved the submitted manuscript.

Jesús M González–Santiago. Hospital Clínico Universitario de Salamanca. Instituto de Investigación Biomédica de Salamanca (IBSAL). Salamanca SPAIN Acquired data, critically reviewed the manuscript drafts, and approved the submitted manuscript.

Santiago Frago Larramona. Hospital Santa Bárbara, Soria, SPAIN. Acquired data, critically reviewed the manuscript drafts, and approved the submitted manuscript.

Antonio Cuadrado Lavín. Hospital Universitario Marqués de Valdecilla, Santander, SPAIN. Acquired data, critically reviewed the manuscript drafts, and approved the submitted manuscript.

Marta Lozano Lanagrán. Hospital Quirónsalud, Málaga, SPAIN. Acquired data, critically reviewed the manuscript drafts, and approved the submitted manuscript.

Francisco Javier Zozaya Larequi. Policlínica Gipúzkoa, San Sebastián, SPAIN. Acquired data, critically reviewed the manuscript drafts, and approved the submitted manuscript.

Antonio Cerezo Ruiz. Hospital de Alta Resolución Sierra de Segura, Jaén, SPAIN. Acquired data, critically reviewed the manuscript drafts, and approved the submitted manuscript.

Sandra Agudo Fernández. Hospital Rey Juan Carlos, Móstoles, Madrid, SPAIN. Acquired data, critically reviewed the manuscript drafts, and approved the submitted manuscript.

Lorena Sancho del Val. Hospital Rey Juan Carlos, Móstoles, Madrid, SPAIN. Acquired data, critically reviewed the manuscript drafts, and approved the submitted manuscript.

Angel Cedeño Zambrano. Hospital Comarcal Sant Jaume de Calella, Barcelona, SPAIN. Acquired data, critically reviewed the manuscript drafts, and approved the submitted manuscript.

Leyanira Torrealba Medina. Hospital Universitari de Girona Doctor Josep Trueta, Girona, SPAIN. Acquired data, critically reviewed the manuscript drafts, and approved the submitted manuscript.

Figure S1. Regional distribution of Spanish centres participating in the Hp–EuReg.

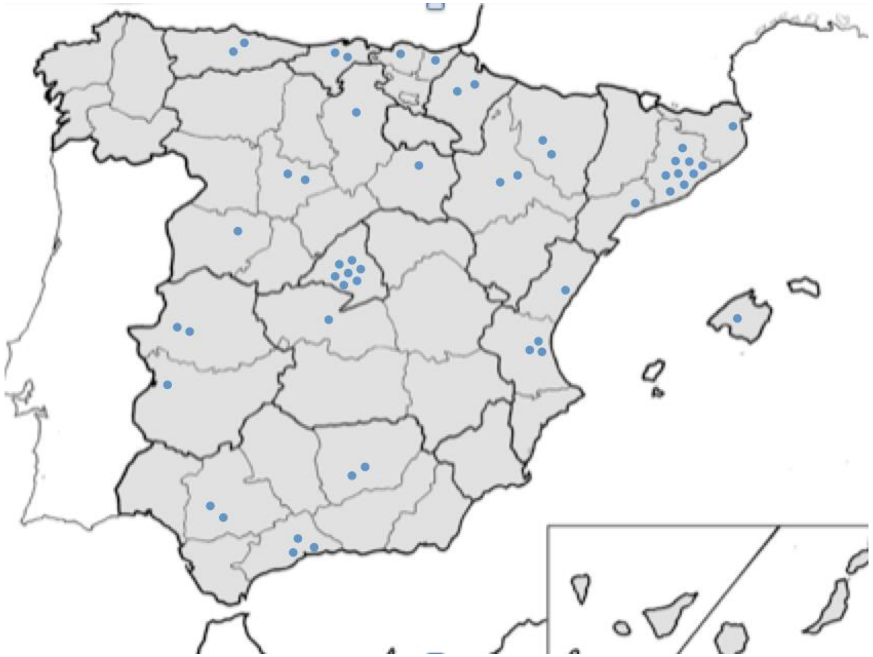

Table S1. Safety in first– and second–line treatment.

	Adverse events			
	1st line		*2nd line*	
**Intensity**	N (%)	95% CI	N (%)	95% CI
**Mild**	5,126 (62)	61–63	1,434 (47)	45–50
**Moderate**	5,126 (34)	33–36	1,434 (49)	46–52
**Severe**	5,126 (3.8)	3.2–4.3	1,434 (3.8)	2.9–5
**Serious***	5,126 (0.1)	0.03–0.23	1,434 (0.07)	0.002–0.4

95% CI: 95%confidence interval, *Serious adverse event: event leading to hospitalization, disability, or death, or to birth defects on pregnant patients.

Table S2. Effectiveness in first– and second–line treatment according to duration.

**Effectiveness**	**ITT**						**mITT**						**PP**					
**Days**	**7**		**10**		14		7		10		14		7		10		14	
**T**	**N (%)**	**95% CI**	**N (%)**	**95% CI**	N (%)	95% CI	N (%)	95% CI	N (%)	95% CI	N (%)	95% CI	N (%)	95% CI	N (%)	95% CI	N (%)	95% CI
First line	159 (60)	52–68	6,237 (83)	83–84	3,668 (85)	84–86	159 (60)	52–68	6,011 (88)	87–89	3,522 (90)	89–91	158 (61)	53–68	5,858 (89)	88–90	3,449 (90)	89–91
**PPI + C + A + M (Conc)**	4 (100)	40–100	2,323 (85)	83–86	1,654 (88)	86–90	4 (100)	40–100	2,232 (88)	87–90	1,629 (92)	91–93	4 (100)	40–100	2,175 (89)	88–90	1,588 (92)	91–94
**PPI + C + A**	146 (59)	51–67	1,767 (80)	78–82	784 (77)	74–80	146 (59)	51–67	1,686 (84)	82–86	699 (86)	84–89	145 (59)	51–67	1,657 (84.5)	83–86	683 (87)	84–89
**PPI + Single–capsule**	2 (100)	16–100	1,566 (88)	86–89	3 (67)	9–99	2 (100)	16–100	1,533 (95)	94–96	2 (100)	16–100	2 (100)	16–100	1,507 (96)	95–97	2 (100)	16–100
**PPI + Bi + C + A**	0 (0)	NA	10 (80)	44–98	1,022 (88.5)	86–90	0 (0)	NA	9 (89)	52–100	1,004 (91)	89–93	0 (0)	NA	8 (100)	63–100	992 (91)	89–93
**PPI + C + A + M (Seq)**	0 (0)	NA	229 (79)	73–84	1 (100)	NA	0 (0)	NA	221 (81)	76–86	1 (100)	NA	0 (0)	NA	191 (84)	78–89	1 (100)	NA
**PPI + C + M**	2 (100)	16–100	89 (58)	48–69	33 (58)	39–75	2 (100)	16–100	82 (63)	52–74	29 (65.5)	46–82	2 (100)	16–100	81 (64)	53–75	29 (65.5)	46–82
Second line	17 (71)	44–90	1,345 (74)	72–76	1,051 (85)	83–87	17 (71)	44–90	1,265 (79)	77–81	1,007 (89)	87–91	17 (71)	44–90	1,241 (80)	77–82	986 (90)	88–92
**PPI + L + A**	4 (75)	19–99	680 (70)	66–73	259 (86)	81–90	4 (75)	19–99	647 (74)	70–77	241 (92)	88–95	4 (75)	19–99	636 (74)	70–77	240 (92.5)	88–96
**PPI + Bi + L + A**	0 (0)	NA	8 (50)	16–84	454 (87)	83–90	0 (0)	NA	6 (67)	22–96	444 (90)	86–92	0 (0)	NA	6 (67)	22–96	428 (90)	87–93
**PPI + Single–capsule**	0 (0)	NA	432 (80)	76–84	7 (86)	42–100	0 (0)	NA	399 (88.5)	85–91	7 (67)	22–96	0 (0)	NA	390 (89)	86–92	7 (67)	22–96
**PPI + Mx + A**	0 (0)	NA	21 (95)	76–100	114 (85)	77–91	0 (0)	NA	20 (100)	83–100	109 (89)	82–94	0 (0)	NA	20 (100)	83–100	109 (89)	82–94
**PPI + C + A + M (Conc)**	0 (0)	NA	51 (74.5)	60–86	68 (75)	63–85	1 (0)	NA	47 (81)	67–91	62 (84)	72–92	1 (0)	NA	46 (80)	66–91	62 (84)	72–92

ITT: intention–to–treat, mITT: modified intention–to–treat, PP: per protocol, T: treatment prescribed, N: number of patients included in the analysis, %: proportion of patients presenting effectiveness, 95% CI: 95% confidence interval, PPI: proton pump inhibitor, C: clarithromycin, A: amoxicillin, M: metronidazole, Single–capsule: three–in–one single capsule, Bi: bismuth, L: levofloxacin, Tc: tetracycline, Conc: concomitant administration of the drugs, Seq: sequential administration of the drugs, NA: not applicable.

Table S3. Effectiveness in first– and second–line treatment according to the PPI dose.

**Effectiveness**	**ITT**						**mITT**						**PP**					
**PPI doses**	**Low**		**Standard**		High		Low		Standard		High		Low		Standard		High	
T	N (%)	95% CI	N (%)	95% CI	N (%)	95% CI	N (%)	95% CI	N (%)	95% CI	N (%)	95% CI	N (%)	95% CI	N (%)	95% CI	N (%)	95% CI
1st line	3708 (79.5)	78–81	2570 (84.5)	83–86	3696 (87)	86–88	3536 (84)	83–85	2464 (89)	88–90	3608 (92)	91–93	3421 (85)	84–86	2421 (89.5)	88–91	3537 (92)	91–93
**PPI + C + A + M (Conc)**	1705 (84)	82–86	760 (86)	84–89	1506 (89)	87–91	1623 (89)	87–90	744 (89)	86–91	1492 (92)	91–94	1574 (89)	87–91	731 (89)	87–91	1455 (93)	91–94
**PPI + C + A**	927 (70)	67–73	1114 (82)	80–84	639 (83)	80–86	880 (74)	71–77	1,049 (87)	85–89	584 (91)	89–93	866 (74)	71–77	1,032 (87)	85–89	569 (92)	89–94
**PPI + Single–capsule**	657 (87)	84–89	418 (86)	86–89	473 (91)	88–93	632 (93.5)	91–95	398 (95)	93–97	484 (97)	95–98	621 (94)	92–96	388 (96)	93–98	479 (97)	95–98
**PPI + Bi + C + A**	15 (87)	60–98	120 (97)	92–99	890 (88)	85–90	16 (82)	54–96	120 (97)	92–99	873 (91)	89–93	15 (87)	60–98	119 (97)	92–99	862 (91)	89–93
**PPI + C + A + M (Seq)**	187 (79)	72–84	12 (92)	62–100	31 (74)	55–88	179 (82)	76–87	12 (92)	62–100	31 (74)	55–88	149 (86)	79–91	12 (92)	62–100	31 (74)	55–88
**PPI + C + M**	59 (52.5)	39–66	43 (70)	54–83	22 (54.5)	32–76	58 (53)	40–67	39 (77)	61–89	16 (75)	48–93	57 (54)	41–68	39 (77)	61–89	16 (75)	48–93
2nd line	731 (71)	68–75	451 (74)	70–78	1,207 (85)	83–87	683 (77)	73–80	421 (79)	75–83	1,162 (89)	87–91	670 (77)	74–80	414 (80)	76–84	1,135 (90)	88–91
**PPI + L + A**	388 (68)	63–73	259 (71)	65–77	291 (85)	81–89	365 (72)	67–77	245 (75)	69–80	278 (89)	85–93	361 (72)	67–77	239 (76)	70–81	276 (89.5)	85–93
**PPI + Bi + L + A**	25 (60)	39–79	15 (87)	60–98	416 (88)	84–91	20 (75)	51–91	15 (87)	60–98	410 (90)	87–93	20 (75)	51–91	15 (87)	60–98	394 (90)	88–94
**PPI + Single–capsule**	198 (80)	74–85	72 (81)	70–89	165 (80)	73–86	184 (87)	81–92	65 (91)	81–97	152 (89)	83–93	180 (88)	83–93	65 (91)	81–97	146 (89)	83–94
**PPI + Mx + A**	3 (100)	29–100	18 (94)	73–100	114 (85)	77–91	3 (100)	29–100	17 (100)	81–100	109 (89)	82–94	3 (100)	29–100	17 (100)	81–100	109 (89)	82–94
**PPI + C + A + M (Conc)**	35 (80)	63–92	17 (59)	33–82	68 (75)	63–85	34 (82)	66–93	13 (77)	46–95	63 (82.5)	71–91	33 (82)	65–93	13 (77)	46–95	63 (82.5)	71–91

ITT: intention–to–treat, mITT: modified intention–to–treat, PP: per protocol, T: treatment prescribed, N: number of patients included in the analysis, %: proportion of patients presenting effectiveness, PPI: proton pump inhibitor, Low: ≈ 20 mg omeprazole equivalents b.i.d., Standard: ≈ 40 mg omeprazole equivalents b.i.d., High: ≈ 60 mg omeprazole equivalents b.i.d., 95% CI: 95% confidence interval, C: clarithromycin, A: amoxicillin, M: metronidazole, Single–capsule: three–in–one single capsule, Bi: bismuth salts, L: levofloxacin, Tc: tetracycline., Conc: concomitant administration of the drugs, Seq: sequential administration of the drugs.

Table S4. Effectiveness, safety and compliance of the most frequent first– and second–line therapies prescribed to penicillin–allergic patients.

	Effectiveness						Adverse events		Compliance	
	ITT		mITT		PP					
**T**	N (%)	95% CI	N (%)	95% CI	N (%)	95% CI	N (%)	95% CI	N (%)	95% CI
1st line	408 (76)	71–80	384 (81)	77–85	376 (82)	78–86	393 (24)	20–28	394 (97)	94–98
**PPI + Single–capsule**	151 (85)	79–91	141 (94)	88–97	136 (95)	90–98	143 (27)	20–35	144 (94)	89–98
**PPI + C + M**	117 (60)	50–69	109 (64)	55–73	109 (64)	55–73	114 (15)	9–23)	113 (99)	95–100
**PPI + C + L**	38 (79)	63–91	37 (81)	65–92	36 (83)	67–94	37 (24)	12–41)	37 (97)	86–100
**PPI + Tc + Bi + M**	34 (85)	69–95	33 (88)	72–97	33 (88)	72–97	34 (38)	22–56	34 (100)	90–100
2nd line	136 (73.5)	65–81	131 (77)	69–84	125 (79)	71–86	134 (36)	28–45	134 (94)	89–97
**PPI + Single–capsule**	34 (71)	53–85	33 (73)	55–87	30 (80)	61–92	32 (28)	14–47	32 (94)	79–99
**PPI + C + L**	29 (69)	49–85	26 (77)	56–91	25 (76)	55–91	29 (24)	10–44)	29 (86)	68–96
**PPI + Tc + Bi + M**	21 (90.5)	70–99	20 (95)	75–100	20 (95)	75–100	21 (90.5)	70–99	21 (100)	84–100

ITT: intention–to–treat, mITT: modified intention–to–treat, PP: per protocol, T: treatment prescribed, N: number of patients analized for each variable, %: proportion of patients presenting the variable (success, adverse events o good compliance), 95% CI: 95% confidence interval, PPI: proton pump inhibitor, Single–capsule: three–in–one single capsule, C: clarithromycin, M: metronidazole, L: levofloxacin, Tc: tetracycline, Bi: bismuth.

Table S5. Effectiveness in first– and second–line treatment according to duration in penicillin–allergic patients.

**Effectiveness**	**ITT**						**mITT**						**PP**					
**Days**	7		10		14		7		10		14		7		10		14	
**T**	N (%)	95% CI	N (%)	95% CI	N (%)	95% CI	N (%)	95% CI	N (%)	95% CI	N (%)	95% CI	N (%)	95% CI	N (%)	95% CI	N (%)	95% CI
1st line	5 (60)	15–95	335 (78)	73–82	67 (69)	56–79	5 (60)	60(15–95)	315 (83.5)	79–87	63 (73)	60–83	5 (60)	15–95	308 (84)	80–88	63 (73)	60–83
**PPI + Single–capsule**	NA	NA	150 (85)	79–91	1 (100)	NA	NA	NA	140 (94)	88–97	1 (100)	NA	NA	NA	135 (95)	90–98	1 (100)	NA
**PPI + C + M**	2 (100)	16–100	84 (58)	47–69	31 (61)	42–78	2 (100)	16–100	78 (63)	51–74	29 (65.5)	46–82	2 (100)	16–100	78 (63)	51–74	29 (65.5)	46–82
**PPI + C + L**	NA	NA	33 (79)	61–91	4 (100)	40–100	NA	NA	32 (81)	64–93	4 (100)	40–100	NA	NA	32 (81)	64–93	4 (100)	40–100
**PPI + Tc + Bi + M**	NA	NA	29 (83)	64–94	5 (100)	48–100	NA	NA	28 (86)	67–96	5 (100)	48–100	NA	NA	28 (86)	67–96	5 (100)	48–100
2nd line	1 (100)	NA	94 (67)	57–76	40 (87.5)	73–96	1 (100)	NA	88 (72)	61–81	41 (88)	74–96	1 (100)	NA	84 (74)	63–83	40 (90)	76–97
**PPI + Single–capsule**	NA	NA	33 (70)	51–84	1 (100)	NA	NA	NA	32 (72)	53–86	1 (100)	NA	NA	NA	29 (79)	60–92	1 (100)	NA
**PPI + C + L**	1 (100)	NA	24 (67)	45–84	4 (75)	19–99	1 (100)	NA	21 (76)	53–92	4 (75)	19–99	1 (100)	NA	20 (75)	51–91	4 (75)	19–99
**PPI + Tc + Bi + M**	NA	NA	13 (85)	55–98	8 (100)	63–100	NA	NA	12 (92)	62–100	8 (100)	63–100	NA	NA	12 (92)	62–100	8 (100)	63–100

ITT: intention–to–treat, mITT: modified intention–to–treat, PP: per protocol, T: treatment prescribed, N: number of patients analized for each variable, %: proportion of patients presenting efficacy, 95% CI: 95% confidence interval, PPI: proton pump inhibitor, Single–capsule: three–in–one single capsule, C: clarithromycin, M: metronidazole, L: levofloxacin, Tc: tetracycline, Bi: bismuth, NA: not applicable.

Table S6. Effectiveness in first and second–line treatment according to PPI dose in penicillin–allergic patients.

**E**	**ITT**						**mITT**						**PP**					
**PPI doses**	Low		Standard		High		Low		Standard		High		Low		Standard		High	
**T**	N (%)	95% CI	N (%)	95% CI	N (%)	95% CI	N (%)	95% CI	N (%)	95% CI	N (%)	95% CI	N (%)	95% CI	N (%)	95% CI	N (%)	95% CI
1st line	160 (69)	61–76	128 (83)	75–89	112 (78)	69–85	152 (73)	65–80	122 (87)	80–92	103 (86)	78–92	148 (74)	66–81	120 (87.5)	80–93	101 (87)	79–93
**PPI + Single–capsule**	62 (84)	72–92	31 (90)	74–98	55 (84)	71–92	57 (93)	83–98	28 (100)	88–100	53 (91)	79–97	54 (94)	85–99	27 (100)	87–100	52 (92)	82–98
**PPI + C + M**	55 (53)	39–66	41 (71)	55–84	21 (57)	34–78	55 (53)	39–66	38 (76)	69–89	16 (75)	48–93	55 (53)	39–66	38 (76)	60–89	16 (75)	48–93
**PPI + C + L**	11 (54.5)	23–83	19 (84)	60–97	7 (100)	59–100	10 (60)	26–88	19 (84)	60–97	7 (100)	59–100	9 (67)	30–93	19 (84)	60–97	7 (100)	59–100
**PPI + Tc + Bi + M**	12 (67)	35–90	19 (95)	74–100	3 (100)	29–100	11 (73)	39–94	19 (95)	74–100	3 (100)	29–100	11 (73)	39–94	19 (95)	74–100	3 (100)	29–100
2nd line	49 (61)	46–75	24 (79)	58–93	61 (80)	68–89	44 (68)	52–81	24 (79)	58–93	61 (82)	70–91	41 (73)	57–86	23 (78)	56–93	59 (83)	71–92
**PPI + Single–capsule**	20 (75)	51–91	2 (100)	16–100	12 (58)	28–85	20 (75)	51–91	2 (100)	16–100	11 (64)	31–89	18 (83)	59–96	2 (100)	16–100	10 (70)	35–93
**PPI + C + L**	11 (54.5)	23–83	6 (83)	36–100	11 (73)	39–94	8 (75)	35–97	6 (83)	36–100	11 (73)	39–94	8 (75)	35–97	5 (80)	28–100	11 (73)	39–94
**PPI + Tc + Bi + M**	2 (0)	NA	5 (100)	48–100	14 (100)	77–100	1 (0)	NA	5 (100)	48–100	14 (100)	77–100	1 (0)	NA	5 (100)	48–100	14 (100)	77–100

E: effectiveness, ITT: intention–to–treat, mITT: modified intention–to–treat, PP: per protocol, PPI: proton pump inhibitor, Low: ≈ 20 mg omeprazole equivalents b.i.d., Standard: ≈ 40 mg omeprazole equivalents b.i.d., High: ≈ 60 mg omeprazole equivalents b.i.d., T: treatment prescribed, N: number of patients analized for each variable, %: proportion of patients presenting efficacy, 95% CI: 95% confidence Interval, Single–capsule: three–in–one single capsule, C: clarithromycin, M: metronidazole, L: levofloxacin, Tc: tetracycline, Bi: bismuth, NA: not applicable.
